# Supporting physiological resilience to climate change through anthropology, environmental exercise physiology and sport science: are we doing enough?

**DOI:** 10.1007/s00421-026-06457-6

**Published:** 2026-10-13

**Authors:** Daniel P. Longman, Tadej Debevec, Jan G. Bourgois

**Affiliations:** 1 School of Sport, Exercise and Health Sciences, Loughborough University, Epinal Way, Loughborough, LE11 3TU UK; 2 Faculty of Sport, University of Ljubljana, Gortanova Ulica 22, 1000 Ljubljana, Slovenia; 3 Department of Automatics, Biocybernetics and Robotics, Jožef Stefan Institute, Jamova Cesta 39, 1000 Ljubljana, Slovenia; 4 Department of Movement and Sports Sciences, Faculty of Medicine and Health Sciences, Ghent University, Watersportlaan 2, 9000 Ghent, Belgium; 5 Centre of Sports Medicine, Ghent University Hospital, Corneel Heymanslaan 10, 9000 Ghent, Belgium

**Keywords:** Climate change, Adaptation, Acclimation, Resilience, Physical activity

## Abstract

Climate change poses escalating threats to human physiological function and health. Rising temperatures and increasing climate volatility place growing demands on thermoregulatory, cardiovascular and musculoskeletal systems, yet mitigation cannot prevent near-term impacts of acute climatic events. Complementary strategies to enhance physiological resilience—the capacity to sustain, absorb and recover from physiological stressors to maintain homeostasis—are therefore urgently needed. We argue that insights from anthropology, environmental exercise physiology and sport science offer an integrated framework for understanding and strengthening physiological resilience. Physical inactivity, affecting one-third of adults globally, compounds climate-related vulnerability by eroding the thermoregulatory, cardiovascular and musculoskeletal systems most critical for tolerating environmental stress. An anthropological perspective explains why: throughout human evolution, regular physical activity (PA) was a prerequisite for survival, shaping the biological systems that these disciplines now seek to optimise. Evidence-based strategies to strengthen physiological resilience are reviewed, including endurance and resistance training (which enhance thermoregulatory capacity, cardiovascular function and muscular resilience) and heat acclimation and acclimatisation, which improve thermal tolerance through sudomotor, cardiovascular, fluid regulatory and metabolic adaptations. Cooling strategies, cold habituation and cross-acclimation/acclimatisation offer further means of building resilience. We identify two broad at-risk groups, with particular attention to vulnerability related to ageing and other factors: those who are physically inactive and lack the resilience to sustain, absorb and recover from stressors under climatic extremes, and those who are regularly active but directly exposed to environmental volatility through occupational exposure. We advocate prioritising equitable, community-level implementation to ensure that these benefits are accessible to all.

## Climate change is a pressing physiological challenge

Climate change is one of the defining environmental challenges of the twenty-first century, posing escalating threats to human function and health. Rising mean global temperatures and increasing climate volatility—including more frequent heatwaves, cold snaps, droughts, storms and floods—can disrupt homeostasis, impair function and compromise health both directly (e.g., injury and mortality) and indirectly (e.g., increased infectious disease risk, decreased physical and mental wellbeing and strain on healthcare systems) (Weilnhammer et al. 2021; van Daalen et al. 2024; Bourgois et al. 2026). While large-scale climate change mitigation remains essential, it cannot prevent the near-term physiological impacts of acute climatic events. Complementary strategies are therefore urgently needed to enhance physiological resilience to tolerate climatic extremes.

Here, we argue that insights from anthropology, environmental exercise physiology and sport science offer a framework for understanding physiological resilience and for developing targeted, scalable interventions that strengthen whole body function through physical activity (PA). We define physiological resilience as a dynamic process of mobilising resources at the molecular, cellular, organ and whole-body level to sustain, absorb and recover from internal and external stressors to return to homeostasis or to a new homeostatic level (Blum 2016; Jatobá 2025; Bourgois et al. 2026). This interdisciplinary perspective has the potential to inform both individual- and population-level strategies to bolster resilience under climatic stress and identify at-risk groups.

## The compounding effects of widespread physical inactivity

Despite the well-established adaptive, physical and mental health benefits of regular PA (Warburton and Bredin 2017), physical inactivity is rising globally. Today, around one-third of adults (~ 1.8 billion people) are insufficiently active, substantially increasing the risk of non-communicable diseases and contributing an estimated $27 billion annually to healthcare costs (WHO 2024). Advances within environmental exercise physiology and sport science reveal how regular PA induces controlled physiological strain, challenging thermoregulatory, cardiovascular and musculoskeletal systems and activating adaptive molecular and cellular pathways that enhance tolerance to environmental extremes (Ordway 2001; Blum 2016; Debevec et al. 2024).

An evolutionary perspective explains why insufficient PA has such profound physiological consequences. For most of human evolutionary history, daily life required sustained physical effort. Humans subsisted by hunting wild animals and gathering wild plants for ~95% of our evolutionary past, beginning with *Homo erectus* ~ 2 million years ago (Aiello and Dean 1990; Hamilton and Walker 2018; Longman et al. 2020; Bellwood 2023). This lifestyle involved high levels of habitual PA, as observed in contemporary hunter-gatherers (Pontzer 2015). Throughout our evolution, regular PA was a requirement for survival—an insight that anthropology uniquely provides and that helps explain why its absence carries such profound physiological consequences.

At the same time, limited and unpredictable food availability led natural selection to favour energy conservation and energetic allocation to functions that directly enhanced reproductive success. Maintaining metabolically expensive systems—such as skeletal muscle, bone and cardiovascular tissue—was advantageous only when regularly used. PA therefore acted as a key biological signal indicating that continued investment in these systems was required. If PA levels decline, these tissues are downregulated to conserve and reallocate energy. In contemporary sedentary environments, the absence of this evolutionary stimulus predictably results in reduced muscle mass, skeletal strength and cardiovascular capacity (Lieberman 2015).

Contemporary physical inactivity therefore compounds the physiological threats posed by climate change. Environmental volatility increases thermo-physiological demands precisely when chronic inactivity is eroding the functional capacity and physiological resilience of those exposed to it. The mechanisms are well established: without regular PA, thermoregulatory efficiency declines, cardiovascular reserve diminishes and musculoskeletal resilience weakens (Ravanelli et al. 2021; Deshayes and Périard 2023)—precisely the systems that must perform under climatic stress. Those thermo-physiological demands are particularly pronounced during physical activity in hot conditions, where activity and heat exposure impose additive strain on the cardiovascular and fluid regulation systems (Périard et al. 2021). To facilitate muscle and cerebral oxygenation alongside evaporative heat loss, blood flow must be competitively redistributed between the working muscles, the brain and the skin. This well-documented “competition” between those systems can prove particularly challenging (Joyner and Casey 2015; Trangmar and González-Alonso 2019). The consequently heightened blood flow demand not only compromises exercise and thermoregulatory capacity but can also significantly challenge systemic blood pressure regulation by resetting baroreflex sensitivity, reducing the baroreflex range and altering muscle sympathetic nerve activity (Crandall 2008; Hart and Charkoudian 2014). Physical inactivity thus not only harms population health directly but also amplifies vulnerability to climate-related stressors, weakening the physiological resilience needed to tolerate a rapidly changing environment.

## Strengthening physiological resilience via physical activity

The physiological adaptations induced by regular PA strengthen the body's ability to maintain homeostasis under climatic extremes. Physical inactivity, by contrast, limits these adaptations, reducing physiological resilience and increasing vulnerability to environmental volatility.

Physiological vulnerability to climate change is not uniform. Two broad at-risk groups can be identified: those who are physically inactive and therefore lack the physiological resilience to tolerate climatic extremes, and those who are regularly active, but whose occupational or recreational circumstances expose them directly to environmental volatility, such as outdoor workers, athletes and military personnel. These groups face distinct but equally pressing challenges: the former must build resilience from a low baseline; the latter must maintain it under sustained environmental stress.

The elderly represent an often neglected but highly vulnerable population in the face of climate change (Meade et al. 2020). Given well-established age-related declines in physical and physiological capacities, the ability to appropriately buffer environmental strain can be significantly compromised and further exacerbated in individuals with chronic diseases, particularly cardiovascular conditions (Falchetta et al. 2024). Resilience to environmental strain is often further eroded by a pattern of reduced activity levels, which ultimately lead to further physical and physiological deconditioning (Cunningham et al. 2020). Together, such systemic deconditioning leads not only to reductions in physical and thermoregulatory capacity but can also reduce baroreflex sensitivity and, thus, worsen orthostatic tolerance, particularly so in the hot conditions.

Sex differences may further compound climate-related vulnerability: women consistently report lower physical activity levels than men across age groups globally (Guthold et al. 2020), potentially reducing baseline physiological resilience to environmental strain. Although women do not appear to be inherently more vulnerable to the physiological effects of heat stress than men (Yanovich et al. 2020; Longman et al. 2021), this disparity in habitual activity levels nonetheless warrants careful consideration when designing climate-resilience interventions.

Vulnerability to climate change is also significantly influenced by socioeconomic status (Singh et al. 2025): low-income, developing countries and socially marginalised communities are disproportionately affected, often lacking the resources and capacity to mitigate and prevent climate-related harms. These vulnerabilities are frequently compounded in rapidly urbanising developing-country cities, where the urban heat-island effect, high levels of air pollution, population density and poor sanitation combine to increase health risk (Campbell-Lendrum and Corvalán 2007; St. Louis and Hess 2008).

Based on the above, it is clear that climate change not only poses heightened risks to healthy (inactive) individuals, but even more so to individuals with chronic diseases and other vulnerable groups. Thus, identifying and implementing appropriate, tailored exercise interventions for all represents a critical public health priority (Febbraio and Pedersen 2026).

Strengthening physiological resilience through PA therefore requires addressing many linked challenges: first, increasing engagement with PA among those who are insufficiently active, and second, optimising physiological adaptations that regular PA induces. To address the first, novel strategies are needed to foster PA enjoyment, a key determinant of long-term participation (Hagberg et al. 2009; Klompstra et al. 2022). While technology, gamification and social interaction have been explored (US Centers for Disease Control and Prevention 2011; Edney et al. 2019), the influence of the physical environment is often overlooked. Anthropological insights suggest that natural settings, which represent the ancestral context of human movement, may offer a scalable and low-cost means of improving endurance performance (Longman et al. 2026) and enhancing enjoyment and motivation for future PA (Putland et al. 2026). Significant structural and economic effort also needs to be directed towards improving access to appropriate strategies and countermeasures in low-income populations, but comprehensively addressing these considerations is beyond the scope of the present Perspective.

Evidence-based guidance from environmental exercise physiology and sport science provides clear strategies for improving key components of physical fitness critical for maintaining function in adverse environments (Vollaard et al. 2009; Currier et al. 2023; Jones 2024). While optimal training strategies continue to be refined and individualised, established foundational principles offer a strong basis for public health interventions and can be translated from elite sport to broader populations to inform targeted strategies that enhance climate-related physiological resilience.

Addressing the second challenge—optimising adaptations in those already active—requires the integration of environmental exercise physiology and sport science. Together, these disciplines provide evidence-based strategies to facilitate physiological resilience and manage the compounded strain of physical exertion in an increasingly volatile climate. Physiological resilience can be strengthened through multiple approaches, including training and heat acclimation/acclimatisation, as well as cooling strategies, cold habituation and cross-acclimation/acclimatisation, each considered in turn below, with many approaches applicable regardless of baseline activity level. Endurance training is known to enhance thermoregulatory capacity through increased plasma volume, improved sweating responses and augmented cardiovascular function, with aerobic fitness representing one of the most effective physiological attributes for attenuating rises in core temperature during heat stress (Périard et al. 2015). While this holds true for all age groups, particular focus should be dedicated to maintaining high maximal oxygen consumption (VO_2max_), given its decline with both ageing (Letnes et al. 2023) and with acute heat exposure (Sawka et al. 1985). Combining endurance training with heat acclimation and acclimatisation, for example (and discussed below), may therefore offer particular benefits for heat and exercise tolerance in older age. Resistance exercise also enhances heat tolerance by upregulating heat shock protein expression and enhancing muscular resilience against heat-induced cellular damage (Cramer et al. 2022).

Heat acclimation (via simulated environments) or acclimatisation (via natural exposure) induces adaptations that improve thermal tolerance (Debevec et al. 2024). The underlying mechanisms include increased plasma volume, improved fluid and electrolyte ([Na +] and [Cl^−^]) balance, enhanced sweating capacity, thermal gradient and skin blood flow. Collectively, these adaptations lower cardiovascular strain and core temperature, optimise metabolic efficiency and enhance cellular protection (Périard et al. 2021; Gagnon et al. 2026). These thermoregulatory, cardiovascular, fluid regulatory, sudomotor and metabolic adaptations not only improve exercise performance and work capacity in hot conditions but also, importantly, mitigate the risk of various heat illnesses (e.g., heat exhaustion, heatstroke) and related adverse cardiovascular events. Sweat losses during exercise in hot conditions further reduce plasma volume, compounding the cardiovascular strain already imposed by competing skin and muscle blood flow demands (Périard et al. 2021). Ensuring optimal hydration status remains paramount when implementing high thermal load strategies combining exercise and heat to prevent any unwanted hypohydration and electrolyte imbalance (Akerman et al. 2016). Thus, appropriate and individually tailored hydration strategies remain the cornerstone of maintaining health and wellbeing when under heat stress (Périard et al. 2021).

Heat acclimation/acclimatisation protocols typically involve 6–12 daily sessions of 60–90 min, elevating core body temperature by ~1.5 °C (up to 38.5–39.0 °C). Active strategies (e.g., exercise in heat) are most effective, but passive approaches (e.g., post-exercise hot water immersion or sauna use) also offer benefits (Périard et al. 2021; McDonald et al. 2025). Various cooling strategies may also mitigate thermal strain and support health and performance in hot environments (Meade et al. 2024). Such cooling strategies and protective behaviours are particularly valuable for individuals whose circumstances expose them directly to heat stress (Meade et al. 2020; Chaseling et al. 2025).

Climate change is also increasing the frequency of extreme cold air/water events. While humans can adapt to both heat and cold, our evolved capacity for cold adaptation is generally weaker (Taylor 2014; Ocobock 2023). Although cold acclimation and acclimatisation have been investigated—and some adaptive responses shown [e.g. alterations in brown adipose tissue activity (Yoneshiro et al. 2025)]—effective and scalable population-level protocols remain elusive. Currently, cold habituation, defined as a reduction in an organism's physiological or perceptual response to repeated cold stimuli and typically achieved within five to ten days, represents the most practical approach (Yurkevicius et al. 2022). Behavioural strategies, including appropriate clothing selection, deliberate activity pacing and management of microclimate exposure, also play an important role in mitigating different forms of thermal stress (heat, cold, cold-water immersion) (Bourgois et al. 2026).

Another promising area for supporting climate-change related resilience is cross-acclimation/acclimatisation, wherein adaptation to one environmental stressor improves tolerance to another through shared molecular signalling and protective pathways (Hessemer et al. 1986). While some studies report physiological and functional improvements—such as improved aerobic capacity in the heat following hypoxic exposure—others observe molecular and cellular level changes (e.g., activation of hypoxia-inducible factors and heat shock proteins) without clear functional benefits (Sotiridis et al. 2018, 2022). Interestingly, significantly more focus has been given to employing heat adaptation to optimise hypoxic performance and adaptation (Gibson et al. 2017; Sotiridis et al. 2019; Rodrigues et al. 2026) with the latest meta-analysis on the topic suggesting some benefits although the evidence remains ambiguous (Willmott et al. 2024). Recent observations also suggest that residing at moderate altitude may confer reciprocal benefits through reduced thermal load and possible cardiovascular protection (Burtscher et al. 2025; Mallet et al. 2025). Cold exposure has also been proposed to facilitate cross-adaptation to other environmental stresses, though empirical evidence remains limited (Lunt et al. 2010; Gibson et al. 2017). Collectively, these observations indicate that multiple cross-acclimation/acclimatisation pathways exist but remain insufficiently explored. This is highly relevant for the above-mentioned vulnerable populations, who might particularly benefit from the cardiovascular (Périard et al. 2021) and haematological (Jenkins et al. 2025) effects of such interventions. Strategically applying multiple environmental stressors—sequentially or concomitantly (Girard et al. 2024; Huang et al. 2026)—may therefore offer a means of improving physiological resilience to environmental extremes, though robust and ecologically relevant research is needed to realise this potential.

## Future directions and perspectives

**Fig. 1:**
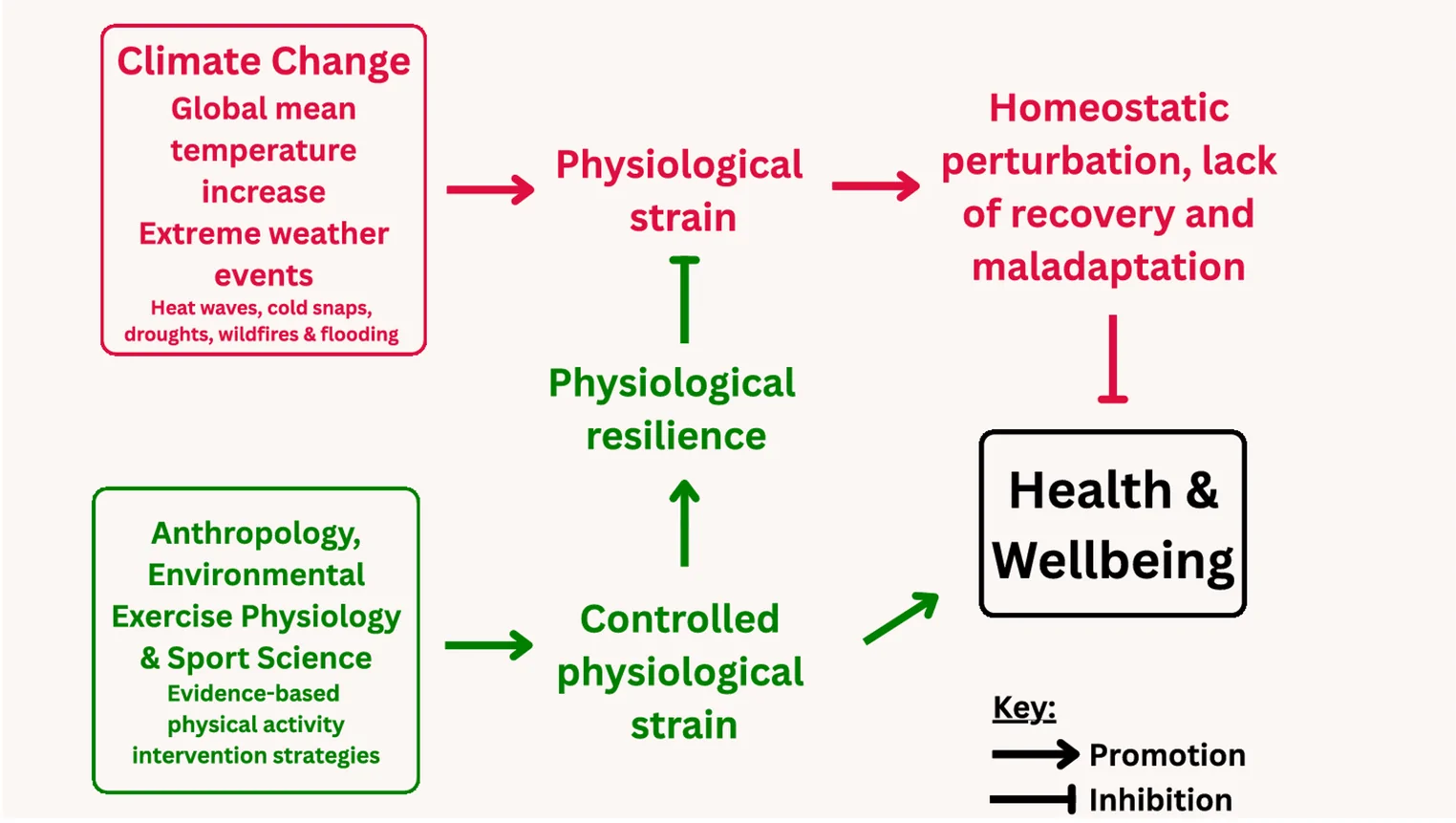
Conceptual framework depicting the escalating physiological threats associated with climate change, driven by rising global mean temperatures and increasing climate volatility, including increasingly frequent extreme weather events (e.g., heat waves, cold snaps, droughts, wildfires and flooding). These environmental stressors increase physiological strain, which, if unresolved, leads to homeostatic perturbation and impaired function, compromising health and wellbeing. Integrating perspectives from anthropology, environmental exercise physiology and sport science provides a framework to strengthen physiological resilience via evidence-based physical activity interventions that induce controlled physiological strain, buffering against climate-related strain and promoting health and wellbeing.

Anthropogenic climate change poses a growing threat to human homeostasis, function and health. We advocate a multidisciplinary approach integrating anthropology, environmental exercise physiology and sport science to develop interventions that enhance physiological resilience. Key priorities include:

- Increasing population-level PA engagement, with priority given to scalable, low-cost strategies, such as nature-based approaches (e.g., green and blue space exercise interventions) that leverage the ancestral context of human movement (Todorova et al. 2023; Longman and Shaw 2026; Putland et al. 2026; Longman et al. 2026).
- Enhancing physiological resilience by:
  o Augmenting evidence-based PA interventions to improve whole body function and physiological resilience under climatic stress.
  o Refining pre-acclimation/acclimatisation strategies to reduce heat stress susceptibility.
  o Integrating active and passive heat acclimation and acclimatisation, and cold habituation protocols, to support whole body function.
  o Developing individualised cooling strategies in hot environments.
  o Developing behavioural strategies (e.g. thermal clothing, activity pacing and microclimate management) to mitigate thermal stress across heat, cold and cold-water immersion.
- Leveraging cross-acclimation/acclimatisation by applying multiple environmental stressors to enhance tolerance to diverse physiological challenges, prioritising scalable, community-level approaches that are accessible to those most at risk.
- Identifying at-risk individuals using sport-science-based assessment methods and designing targeted interventions for vulnerable populations (see Fig. 1).

As mentioned earlier, the populations most exposed to climate-related health risks are frequently those with least access to personalised or high-cost interventions, including individuals in low-income and developing countries, where urban environmental exposure and limited infrastructure compound vulnerability. In addition, elderly populations should be considered and targeted particularly carefully to ensure they remain as physically active and healthy as possible and thereby optimally maintain their physiological resilience. Future research and policy must therefore prioritise scalable, equitable and community-level implementation strategies, ensuring that the benefits of enhanced physiological resilience are accessible to all—and not confined to those with the resources to seek individualised solutions.

In conclusion, while many individuals feel powerless in the face of climate change, perceiving its drivers and consequences as too large for meaningful personal action (Aitken et al. 2011), strategies that enhance physiological resilience offer a rare point of individual agency. By improving fitness and physiological resilience, people can take concrete steps to protect themselves—and those around them—from climate-related health risks. Such approaches do not replace the need for systemic mitigation, but they provide an actionable, evidence-informed pathway that empowers individuals to reduce vulnerability in the near term. Realising this potential requires strong interdisciplinary collaboration to refine and expand evidence-based strategies that augment physiological resilience to environmental extremes.
